# Hospital-Related Lineage of USA300 Methicillin-Resistant *Staphylococcus aureus* (MRSA) to Cause Bacteremia in Iran

**DOI:** 10.1155/2023/8335385

**Published:** 2023-04-15

**Authors:** Masoumeh Navidinia, Samira Zamani, Anis Mohammadi, Shahram Araghi, Chakameh Amini, Behzad Pourhossein, Mehdi Goudarzi

**Affiliations:** ^1^Department of Medical Laboratory Sciences, School of Allied Medical Sciences, Shahid Beheshti University of Medical Sciences, Tehran, Iran; ^2^Department of Microbiology, School of Medicine, Shahid Beheshti University of Medical Sciences, Tehran, Iran; ^3^Department of Microbiology, Faculty of Advanced Science and Technology, Tehran Medical Sciences, Islamic Azad University, Tehran, Iran; ^4^Department of Microbiology, Faculty of Biological Sciences, North Tehran Branch, Islamic Azad University, Tehran, Iran; ^5^Pharmaceutical Sciences Research Center, Shahid Beheshti University of Medical Sciences, Tehran, Iran

## Abstract

*Staphylococcus aureus* is an important pathogen that causes bloodstream infections. This study is aimed at assessing the genotypic characteristics of *S. aureus* strains responsible for bloodstream infections. An epidemiological study was conducted using 85 *S. aureus* strains isolated from bloodstream infections. Susceptibility was tested using the broth microdilution method and disk diffusion. All detected methicillin-resistant *S. aureus* (MRSA) isolates were confirmed by mecA PCR assays. *S. aureus* isolated from bacteremia were characterized using SCCmec, spa, and multilocus sequence typing methods. The prevalence of *S. aureus* strains responsible for bloodstream infections was 38.8%. All isolates were MRSA. Multidrug resistance (MDR) was present in 84.7% of isolates. MRSA isolated categorized within six clonal complexes including CC8 (60%), CC22 (22.4%), CC5 (5.9%), CC30 (4.7%), CC45 (4.7%), and CC59 (2.3%). The main lineages found were USA300/CC8-MRSA-IV/t008 (41.2%), followed by ST22-SCC*mec*IV/t790 (9.4%), ST239-SCC*mec*III/t037 (7.1%), ST22-SCC*mec*IV/t032 (7.1%), ST239-SCC*mec*III/t631 (5.9%), ST239-SCC*mec*III/t860 (5.9%), ST22-SCC*mec*IV/t852 (5.9%), ST5-SCC*mec*IV/t002 (4.7%), ST45-SCC*mec*IV/t038 (4.7%), ST30-SCC*mec*IV/t318 (4.7%), ST59-SCC*mec*IV/t437 (2.3%), and ST225-SCC*mec*II/t045 (1.1%). Resistance to vancomycin amounted to 5.9% of isolates that belonged to ST239-SCC*mec*III/t037 (80%) and ST8-SCC*mec*IV/t008 (20%). The emergence of USA300 strains in bloodstream infections in our country is a serious alarm and highlights the significant invasion of this lineage into the healthcare system. MDR patterns among these strains appear to be becoming the biggest problem in healthcare treatment.

## 1. Introduction

*Staphylococcus aureus* is an important nosocomial pathogen which is known to be responsible for bloodstream infections (BSI) [1]. Considering the recently reported evidence, *S*. *aureus* bacteremia (SAB) is associated with high morbidity and mortality in hospitals. Therefore, several researchers have recently focused on understanding the genotypes of SAB that are responsible for both virulence and antibiotic resistance [2]. Historically, it is well understood that the pathogenesis of SAB is linked to the expression of various virulence and antibiotic resistance determinants [1, 2]. However, in recent decades, certain genotypes of SAB have been documented to cause significant mortality rates, complications, and healthcare costs and may play a critical role in the pathogenesis of *S. aureus*, especially in patients with underlying diseases [2]. Reports on BSI indicate that methicillin-resistant *S. aureus* (MRSA) is one of the most prevalent pathogens that pose a public health challenge and remains highly prevalent in Iran [3, 4].

Over recent years, the dissemination of various *S. aureus* clones has been described in healthcare setting environments. USA300 (ST8-SCC*mec*IV/t008) is a pathogen found in North America in the general population and hospitals and is characterized by its wide genetic diversity, rapid dissemination, and association with invasive infections, such as hematogenous complications and bacteremia [1, 5]. Literature has indicated that USA300 was isolated worldwide and particularly predominant in Spain, Canada, Switzerland, Australia, Iran, and the UAE [6]. USA300 was an infrequent type in Iran. Following efforts made to map out the identification and genotypes of SAB, we have previously indicated a relatively high prevalence of USA600 isolates in BSI which accounted for 29.8% of isolates [4]. In general, reports of USA300 in patients with bacteremia indicated the potential for these strains to spread in hospitals and cause invasive infections. This lineage had high persistence rates in blood, which may be closely associated with hematogenous complications and increased patient mortality [7]. The high likelihood of USA300 becoming epidemiologically prevalent in Iran prompted us to understand its characteristics. Our study attempts to understand the molecular characteristics, antibiotic resistance, and prevalence of *S*. *aureus* strains isolated in blood samples.

## 2. Material and Methods

### 2.1. Bacteria Isolation

The 85 *S. aureus* recovered from BSIs were obtained from 219 blood culture samples during the period of 1.5 years from June 2020 to November 2021. The distribution of the *S. aureus* obtained from investigated hospitals was as follows: 25 isolates (29.4%) from hospital A (Taleghani), 31 isolates (36.5%) from hospital B (Imam Hossein), and 29 isolates (34.1%) from hospital C (Shohad-e-Tajrish). All blood cultures were subjected to bacteriological and biochemical techniques to presumptive detection of *S. aureus*. PCR assay confirmed isolates by detecting the *nuc*A gene, as described earlier [3]. For the *nuc*A gene, the 237 bp amplicons were detected after 1.8% agarose gel electrophoresis and staining with ethidium bromide [4]. Finally, *S. aureus* isolates were stored in 20 percent glycerol tryptic soy broth (TSB; Merck, Darmstadt, Germany) at −70°C to start the laboratory investigation. The research was ethically confirmed by the Ethics Committee of the Shahid Beheshti University of Medical Sciences in Tehran, Iran (IR.SBMU.RETECH.REC.1400.587); also, consent was obtained from participants.

### 2.2. Determination of Isolate Susceptibility

For evaluating isolates' susceptibility against cefoxitin (to identify methicillin resistance), penicillin, nitrofurantoin, clindamycin, rifampin, quinupristin-dalfopristin, erythromycin, ciprofloxacin, linezolid, gentamicin, trimethoprim-sulfamethoxazole, and tetracycline, the Kirby Bauer disk diffusion method was performed based on the 2021 guidelines of the Clinical and Laboratory Standards Institute (CLSI) [8]. Broth microdilution test (Sigma-Aldrich, St. Louis, Mo) was used to estimate the MIC (minimum inhibitory concentration) of mupirocin and vancomycin. ICR (inducible clindamycin resistance) was used to determine the test of the D-zone. *Staphylococcus aureus* isolates with MIC value ≥ 512 *μ*g/mL were measured as HLMUPR (high-level mupirocin resistance) isolates and isolates with MIC range between 8 and 256 *μ*g/mL as low-level and resistance (LLMUPR) isolates, respectively [8]. For screening of MRSA isolates, the *mec*A gene of the bacteria was targeted. An experiment was controlled using reference strains (ATCC 25923, ATCC 43300, and ATCC 29213).

### 2.3. Extraction of Genomic DNA and Detection of Toxin Genes

The phenol-chloroform method was used to extract the total DNA of *S. aureus* strains. Nonodrope was used to check the DNA purity. The detection of genes, *etb* (encoded exfoliative toxin), *pvl* (encoded Panton-Valentine leukotoxin), *eta*, and *tst* (encoded toxic shock syndrome toxin), was performed using a PCR protocol described by Goudarzi et al. [4].

### 2.4. Genotypic Characterization

#### 2.4.1. SCC*mec* Typing

For SCC*mec* typing, the multiplex PCR was performed based on the oligonucleotide sequences and conditions designated by Boye et al. [9]. Obtained banding patterns were analyzed and compared with the banding patterns of the reference stains (WIS (SCC*mec* type V), ATCC 10442 (SCC*mec* type I), MW2 (SCC*mec* type IVa), 85/2082 (SCC*mec* type III), and N315 (SCC*mec* type II)).

#### 2.4.2. *spa* Typing

*spa* typing to analyze the surface protein A was carried out via PCR [10] following the same conditions mentioned earlier. The purification of PCR products using the commercial kit (QIAquick PCR Purification) and analysis of nucleotide sequence performed using an ABI Prism 377 automated sequencer (Applied Biosystems, Perkin-Elmer Co., Foster City, CA) were done. Sequence editing was done using the Chromas software (version 1.45, Australia). *spa* types were determined by submitting edited sequences to the Ridom SpaServer database (http://www.spaserver.ridom.de).

#### 2.4.3. Multilocus Sequence Typing (MLST)

MLST assay is used to identify sequence types (STs) according to internal fragment sequencing (*tpi*A, *arc*C, *gm*K, *glp*F, *aro*E, *yqi*L, and *pta*) and submission of their sequences to the online MLST database website (https://pubmlst.org/). A CC/ST8-SCC*mec*IV/t008 strain was considered USA300 if the ACME (arginine catabolic mobile element) elements were reported as previously explained by Tayebi et al. [7].

## 3. Results

### 3.1. Isolation and Screening of Toxigenic Strains

In our study, 219 blood culture samples were cultured for isolation of *S. aureus*. Of those, 85 (38.8%) were positive comprising 28 (32.9%) male and 57 (67.1%) female. The mean age of patients were 48 years (range, 15-71 years). Patients in the age range of 41-50 years were the most common age category accounted for 37.6%. More than 60% of the patients had the history of hypertension and also previous antibiotic usage for the last 3 months. Demographic data, hospitalization's ward, and risk factors are described in Table 1. Regarding toxigenic isolates, the highest toxin production rate was recorded for PVL (47.1%; 40/85), followed by TST (24.6%, 12/85) and ETa (5.9%, 5/85).

### 3.2. *S. aureus* Bacteremia Antimicrobial Resistance Profiles

Based on the disk diffusion assay, study isolates had cefoxitin resistance and were confirmed as MRSA. Overall, none of the isolates tested for antibacterial susceptibility were susceptible to all of the drugs. Linezolid was the main antibiotic to which all isolates were susceptible and resistant to penicillin. The resistance rate of erythromycin was the highest (84.7%, 72/85) followed by tetracycline (76.5%, 65/85), ciprofloxacin (61.2%, 52/85), gentamicin (55.3%, 47/85), clindamycin (52.9%, 45/85), trimethoprim-sulfamethoxazole (40%, 34/85), mupirocin (36.5%, 31/85), rifampin (35.3%, 30/85), nitrofurantoin (34.1%, 29/85), quinupristin-dalfopristin (20%, 17/85), and vancomycin (5.9%, 5/85). In total, 84.7% (72/85) of isolates were found to be multidrug-resistant (MDR).

**Figure 1:**
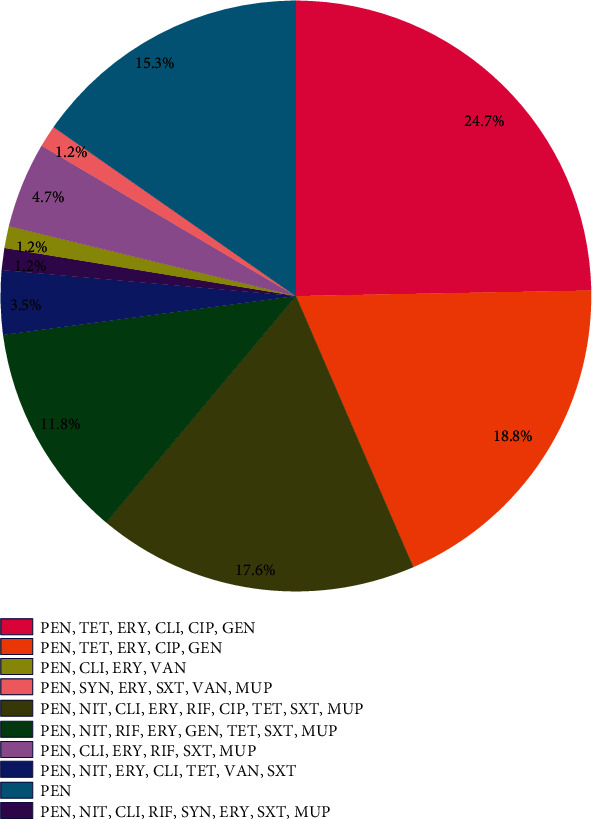
Distribution of antibiotic resistance profiles in MRSA related to bloodstream infection.

Figure 1 shows resistance profiles, wherein CLI, TET, PEN, ERY, CIP, GEN (24.7%, 21/85); PEN, TET, CIP, GEN, ERY (18.8%, 16/85); and PEN, TET, CIP, RIF, NIT, ERY, CLI, SXT, MUP (17.6%, 15/85) were the top three frequently detected profile. Vancomycin microbroth dilution results displayed that 22.4% of the isolates inhibited at MIC value 0.5 *μ*g/mL, 38.8% at 1 *μ*g/mL, 32.9% at 2 *μ*g/mL, and 5.9% at 16 *μ*g/mL. We identified inducible clindamycin resistance (ICR) and constitutive clindamycin resistance (CCR) in 27 (31.8%) and 41 (48.2%) of the isolates, respectively. As previously mentioned in the present study, 36.5% of isolates were confirmed as mupirocin-resistant, of which 10 (11.8%) for HLMUPR and 21 (24.7%) for LLMUPR exhibited the phenotypes.

### 3.3. Molecular Characterization

The SCC*mec* typing results showed that the IV type was highly prevalent representing 80% (68/80) followed by types III (18.8%, 16/85) and SCC*mec* types II (1.2%, 1/85). Based on the *spa* typing method, isolates were allocated to particular t008, t790, t032, t037, t631, t860, t852, t002, t318, t437, and t045 *spa* types. MLST assay detected 8 STs among the tested isolates, namely, USA300/ST8 (41.2%, 35/85), ST22 (22.4%, 19/85), ST239 (18.8%, 16/85), USA800/ST5 (4.7%, 4/85), USA600/ST45 (4.7%, 4/85), USA1100/ST30 (4.7%, 4/85), ST59 (2.4%, 2/85), and ST225 (1.2%, 1/85) which were categorized into 6 clonal complexes (CC22, CC5, CC45, CC8, CC30, and CC59). As shown in Table 2, CC8 was higher than other isolates by 60% (51/85); also, other CCs (CC5, CC45, CC22, CC59, and CC30) were 40%. The highly frequent clone was USA300/CC8-MRSA-IV/t008 which accounted for 41.2% (35/85) of MRSA isolates. Out of 5 vancomycin-resistant MRSA isolates, 4 isolates belonged to ST239-SCC*mec*III/t037 (80%) and one isolate belonged to ST8-SCC*mec*IV/t008 (20%). LLMUPR MRSA isolates belonged to ST8-SCC*mec*IV/t008 (76.2%, 16/21) and ST239-SCC*mec*III/t860 (23.8%, 5/21). The mupirocin results showed that half of the HLMUPR isolates belonged to ST239-SCC*mec*III/t631 clone (5 isolates) while the remaining half were detected in ST22-SCC*mec*IV/t790 (3 isolates) and ST30-SCC*mec*IV/t318 (2 isolates) clones. Overall, 12/85 MRSA isolates (14.1%) were TST-positive and 40/85 (47.1%) PVL-positive. TST-positive isolates belonged to three clones including ST239-SCC*mec*III/t037 (5 isolates), ST22-SCC*mec*IV/t790 (4 isolates), and ST5-SCC*mec*IV/t002 (3 isolates). Specifically, PVL-positive isolates belonged to ST8-SCC*mec*IV/t008 (35 isolates), ST30-SCC*mec*IV/t318 (4 isolates), and ST22-SCC*mec*IV/t852 (1 isolate). ICR phenotype was assigned to 5 particular clones as follows: ST8-SCC*mec*IV/t008 (20%, 17/85), ST239-SCC*mec*III/t037 (5.9%, 5/85), ST225-SCC*mec*II/t045 (1.2%, 1/85), ST22-SCC*mec*IV/t790 (3.5%, 3/85), and ST30-SCC*mec*IV/t318 (1.2%, 1/85).

## 4. Discussion

Our analysis revealed several striking findings: (i) genetic variation of *S*. *aureus* in BSI samples, most of which belonged to the USA300 lineage; (ii) occurrence of major clones USA800, USA1100, and USA600 in our hospitals; (iii) report of ST59- SCC*mec*IV/t437 in Iran; (iv) CC8 was found as the only clone among vancomycin-resistant MRSA isolates, and (v) *pvl* genes detected were the most common toxin and associated with CC8, CC30, and CC22. Among the mupirocin-resistant *S. aureus* strains, three main clones were observed: CC22, CC8, and CC30.

Interestingly, the present study confirmed the occurrence of the USA300 lineage, a very prominent MRSA clone, in BSIs [3, 5, 7]. From the literature, USA300 was the most common community-associated type in North America as an epidemic hypervirulent clone and was increasingly encountered in hospitals [5]. This lineage is characterized by the rapidity of its spread, its virulence, and its resistance profile. It is worth highlighting that the spread of the USA300 clone amplified dramatically in Asia, the USA, and Europe [6, 7, 11]. The presence of USA300 lineage has been reported in previous studies from Iran, China, the United Arab Emirates, Spain, the USA, the UK, Australia, Japan, Kuwait, and Switzerland [6]. The development of the lineage in our country reflects the rapid spread of this pandemic clone, which is probably due to importation of USA300 from European or Asian countries. This hypothesis is also supported by the similarities in toxin and resistance profiles and genetic diversity between our USA300 clones and USA300 strains reported in Kuwait and Ireland [11, 12]. Results of antibiotic susceptibility testing performed for USA300 isolate also showed a high resistance rate to mupirocin at a low level and resistance to vancomycin. Variability in resistance within USA300 clones was formerly displayed by several researchers. In a recent study in the USA, it was documented that identified USA300 lineages were resistant to mupirocin and harbored *mupA* [13]. The present study indicated a vancomycin-resistant USA300 isolate. The finding is supported by the previous observation in our country that reported a CC/ST8-SCC*mec*IV/t008 VRSA isolated from a BSI sample [14]. Similarly, in another study on diabetic patients, Tayebi et al. showed four major lineages with the majority of USA300 and some other isolates [7]. This increasing prevalence of USA300 confirms the ability and large global capacity of this successful clone for spreading in different regions of Iran. Resistance to vancomycin among our USA300 clones emphasized that the use of this antibiotic, as a selective pressure agent, should be carefully monitored. Regarding the high prevalence of USA300, the present study suggested the significant penetration of this lineage into the healthcare setting.

CC/ST22-IV-MRSA, namely, epidemic MRSA-15, is one of the most common pathogens reported from different geographical areas. Our results show a high frequency of CC/ST22 (22.4%) as the second most common lineage in SAB, confirming the previous findings of Laham et al. in Palestine, who reported CC22 as the most common CC (42.7%) in MRSA strains isolated from hospitalized patients [15]. Our CC/ST22-MRSA-IV isolates corresponded to three *spa* types including t790, t032, and t852. In a previous study about MRSA isolated from BSIs, this CC was reported as the most frequent clone, with 39.8% [3]. A previous study in Kuwait demonstrated spa types t790, t032, and t852 were noted in MRSA isolates examined between 1992 and 2010 [11]. This CC is mostly reported in Saudi Arabia, Iran, Kuwait, and the UK. Its presence in Iran could be related to the many travels to these countries each year [6]. According to the SCC*mec* types, *pvl* and *tst* genes, researchers have been stating different variants of ST22-IV-MRSA variants. Based on the present results, our CC/ST22 isolates harbored *pvl* (5.3%) and *tst* (21.1%) genes. CC/ST22 PVL-positive isolates have also been described in clinical samples of Iran [3], Ireland [12], and Kuwait [11]. Alarmingly, the high prevalence of this clone plus toxin encoding genes and MDR pattern may be the start of its epidemic in Iran in the future.

Few prevalences reported about CC/ST22-positive *tst* gene are described in the literature. Regarding CC/ST22 TST-positive isolates, we detected *tst* in 14.1% of SAB isolates and 33.3% belonged to CC/ST22. A consistent result was reported by Laham et al. in Palestine. They analyzed 215 *S. aureus* clinical isolates between 2008 and 2012 and indicated the high frequency of the *tst* gene (27.4%) and found a high prevalence in CC22 (70.4%) [15]. It is well established that CC/ST22 isolates can have different virulence markers and antimicrobial resistance phenotypes. The results of the current study suggest a high prevalence of HLMUPR in the ST22-SCC*mec*IV/t790 clone which is similar to the previously reported rate of 39.8% in Iranian hospitals [16].

Multiresistant CC8/ST239 was the third predominant lineage representing 18.8% of the isolates harboring SCC*mec* type III. This clone corresponded to t037 (37.6%), t631 (31.2%), and t860 (31.2%). This clone is mostly reported in the USA, Iran, Kuwait, Ireland, and Saudi Arabia [11, 12, 17]. In this study, more than half of CC8/ST239-MRSA III strains exhibited mupirocin resistance (ST239-SCC*mec*III/t631 related to HLMUPR phenotype and ST239-SCC*mec*III/t860 related to LLMUPR phenotype). Similarly, a previous study from 2005 to 2013 demonstrated CC8/ST239 as the most predominant clone in their research [18]. Other studies in India and Kuwait indicated that HLMUPR phenotypes are mainly found in CC8/ST239 lineage [11, 19]. The results of the current study indicate that all CC8/LLMUPR-MRSA strains are very similar to our earlier report from Iran (word mup2). A study in Kuwait found a high percentage of MDR-CC8/ST239-MRSAIII/t860 clones without resistance to mupirocin [11]. Therefore, the results reflect a propensity for the rapid spread of this clone between hospitals in our area.

In our survey, the data showed 80% (4/5 isolates) of vancomycin-resistant MRSA isolates belonged to ST239-SCC*mec*III/t037 clone. This is supported by the observations of Havaei et al. from Iran which confirmed ST239-SCC*mec*III/t037 isolated from blood as a vancomycin-resistant isolate [20]. Based on the earlier reports from Pennsylvania [21] and New Zealand [22], vancomycin-resistant ST239 was well documented. In a study by Azimian et al., they found that the recovered VRSA isolate (ST1283-SCC*mec*III/t037) had a variant of ST239 [23]. Results of the meta-analysis performed in 2020 depicted an upward trend in VRSA and VISA around the world. Shariati et al. displayed that VISA strains (1.7%) had a higher global rate than VISA strains (1.5%). Likewise, comparing before and after 2010, researchers have reported an increase in VRSA and VISA. Meanwhile, Asian countries especially Iran and India included the highest rates of incidence of VRSA (67%) [24]. This quite high prevalence of VRSA in the two mentioned above countries compared to America/Europe countries can be due to unrestricted and unscheduled administration of antimicrobials, geographic area, level of hygiene, poor health policies, and diverse attitudes towards antimicrobial protocols.

Despite decreasing trend of CC/ST5 lineage from 8% to 2.6% over the years in Iran [17], our attained data highlighted the existence of CC/ST5 in 5.9% of BSA isolates whichcorresponded to ST5-SCC*mec*IV/t002 (USA800) (80%) and ST5-SCC*mec*II/t045 (20%) clones. This emergence has been reported regionally, as in Iran, the UAE, Australia, Kuwait Japan, Ireland, and Korea [6, 11]. In a 2022 study conducted by Augusto et al. on 123 *S. aureus* isolates recovered from a bloodstream in Brazil, three main lineages were USA800/ST5/SCC*mec*IV, ST105, and USA100/SCC*mec*II/ST5 [1]. Similarly, in a study in Chinese hospitals, Song et al. reported a high frequency of ST5-SCC*mec*IV/t002 [25]. These results are consistent with an earlier study by Williamson et al. in New Zealand, in which CC/ST5 isolates were detected, albeit at low levels. They also showed rapid replacement of this clone by ST30-SCC*mec*IV [26]. In contrast to some studies in which HLMUPR was detected in the USA800 clone, our analysis does not show resistance to mupirocin in any of the USA800 isolates.

CC/ST45-MRSA IV, a clone related to USA600, has a large global spread capacity and high survival rates in the blood which can lead to high mortality in BSIs. In our survey, the data showed a low prevalence rate of CC45/ST45-MRSA-IV/t038 clone accounting for 4.7%. Our observations about USA600 lineage are in line with Liang et al.'s research that showed the emergence of ST45 as ranked third in 18.8% of clinical MRSA isolates in China [27]. In a recent research in Kuwait, ST45 was found to be one of the most epidemic lineages [11]. In another research by Ilczyszyn et al., ST45 accounted for 26.1% of *S. aureus* isolates [28]. In another cross-sectional research involved in *S. aureus* isolates from the blood in Iran in 2018, ST45 was the top sequence type among MRSA isolates (29.8%). Although in the present study the frequency of ST45 was at a low level, compared with our previous study in BSI, the above studies support the view that the ST45 clone gradually expanded and became popular in Iran.

In this study, ST30-SCC*mec*IV/t318 clone (USA1100) was found in 4.7% of tested isolates; all were PVL-positive. Also, we observed the HLMUPR phenotype in half of the isolates of this clone while clindamycin resistance potency was detected in three-fourths of isolates. This clone was reported in Taiwan, the UAE, Iran, Europe, Saudi Arabia, and Egypt [6]. In earlier research about the characterization of clindamycin resistance potency of *S. aureus* clones reported from Iran, this clone was more frequent in healthcare settings, with 11.9% of *S. aureus* isolates. They also showed a significant increasing trend from 8% in 2013 to 18.4% in 2018 [17]. CC30/ST30-IV strains carrying *pvl* genes were reported from Kuwait and Ireland [11, 12]. However, in another study, a significant decrease in the frequency of CC30/ST30 from 30% in 2001–2003 to 22% in 2006 was reported. The same finding by Moghadamet et al. reported MRSA strains belonged to two main CC8 and CC30 [29]. In the current research, the data indicated the identification of MDR ST59-SCC*mec*IV/t437 at a low level.

This lineage was earlier reported by several researchers in Iran, Korea, the United Kingdom, Taiwan, Kuwait, Australia, and Ireland [11, 12, 17]. Our findings support the findings of a recent study conducted by Suyun Qian in China in 2017 on 60 *S. aureus* isolated from children which showed the emergence of four major genotypes of MSSA including ST188-t189, ST59-SCC*mec*IV-t437, ST6-t701ST22-t309, and ST5-t002. Meanwhile, they revealed an MDR pattern mainly in the ST59-SCC*mec*IV-t437 lineage [30]. All ST59-SCC*mec*IV-t437 clones displayed ICR phenotype in our findings. In an Irish study in Ireland to find the molecular characterization of *pvl*-positive MRSA (190 isolates) and MSSA (39 isolates), similar results were reported. Shore et al. indicated the existence of ST59-SCC*mec*IV-t437 in 4.7% of PVL-positive MRSA isolates with high frequencies of MDR pattern, especially ICR phenotype.

One limitation is that pulse field gel electrophoresis types were not determined in our study. This is an issue that should be followed up in future research.

## 5. Conclusion

Overall, these studies indicate the genetic diversity of *S. aureus* from BSI through the majority of USA300. The marked ability of the aforementioned clone to disseminate and its rapid spread between hospitals suggests that this may be the beginning of a future epidemic in Iran. The resistance to vancomycin in our study underscores that the use of and resistance to this and other antibiotics, especially mupirocin and clindamycin, should be carefully monitored.

## Figures and Tables

**Table 1:** Demographics and clinical characteristics of patients with *S. aureus* bacteremia.

Study variables	Frequency	Percent (%)
*Gender*		
Male	28	32.9
Female	57	67.1
*Age category (in years)*		
≤20	8	9.4
21-30	11	12.9
31-40	12	14.1
41-50	32	37.7
51-60	12	14.1
≥61 years	10	11.8
*Ward type*		
Infectious	23	27.1
ICU	20	23.5
Oncology	15	17.6
Surgery	14	16.5
Internal	13	15.3
*Risk factors*		
Previous antibiotic therapy in the last 3 months	53	62.4
History of hospitalization in the last year	36	42.4
History of surgery in the last 6 months	22	25.9
Diabetes mellitus	27	31.8
Hypertension	55	64.7

**Table 2:** Distribution of toxin and resistance patterns in different lineages obtained from BSIs.

CC	ST	*spa* (no., %)	SCC*mec* (no., %)	Toxin genes (no., %)	Antibiotic resistance (no., %)	Hospitals (no., %)	Total *N* (%)
CC8	ST8	t008 (35, 100)	IV (35, 100)	*pvl* (35, 100)	PEN, NIT, CLI, ERY, RIF, CIP, TET, SXT, MUP (14, 40)	A (13, 37.1), B (12, 34.3), C (10, 28.6)	35 (41.2)
					PEN, NIT, RIF, ERY, GEN, TET, SXT, MUP (2, 5.7)		
					PEN, CLI, ERY, VAN (1, 2.9)		
					PEN, TET, ERY, CIP, GEN (15, 42.9)		
					PEN (3, 8.5)		
	ST239	t631 (5, 31.3), t037 (6, 37.4), t860 (5, 31.3)	III (16, 100)	*tst* (5, 31.3)	PEN, NIT, ERY, CLI, TET, VAN, SXT (3, 18.7)	A (4, 25), B (7, 43.8), C (5, 31.2)	16 (18.8)
					PEN, SYN, ERY, SXT, VAN, MUP (1, 6.3)		
					PEN, NIT, CLI, RIF, SYN, ERY, SXT, MUP (1, 6.3)		
					PEN, CLI, ERY, RIF, SXT, MUP (4, 25)		
					PEN, NIT, RIF, ERY, GEN, TET, SXT, MUP (4, 25)		
					PEN (3, 18.7)		
CC5	ST5	t002 (4, 100)	IV (4, 100)	*tst* (3, 75)	PEN, TET, ERY, CLI, CIP, GEN (4, 100)	B (1, 25), C (3, 75)	4 (4.7)
	ST225	t045 (1, 100)	II (1, 100)	*eta* (1, 100)	PEN, TET, ERY, CIP, GEN (1, 100)	A (1, 100)	1 (1.2)
CC22	ST22	t790 (8, 42.1), t032 (6, 31.6), t852 (5, 26.3)	IV (19, 100)	*pvl* (1, 5.3), *tst* (4, 21.1)	PEN, NIT, RIF, ERY, GEN, TET, SXT, MUP (3, 15.8)	A (4, 21), B (6, 31.6), C (9, 47.4)	19 (22.4)
					PEN, TET, ERY, CLI, CIP, GEN (11, 57.9)		
					PEN (5, 26.3)		
CC45	ST45	t038 (4, 100)	IV (4, 100)	*eta* (2, 50)	PEN, TET, ERY, CLI, CIP, GEN (4, 100)	A (1, 25), B (3, 75)	4 (4.7)
CC59	ST59	t437 (2, 100)	IV (2, 100)	*eta* (2, 100)	PEN, TET, ERY, CLI, CIP, GEN (2, 100)	B (1, 50), C (1, 50)	2 (2.3)
CC30	ST30	t318 (4,)	IV (4, 100)	*pvl* (4, 100)	PEN, NIT, CLI, ERY, RIF, CIP, TET, SXT, MUP (1, 25)	A (2, 50), B (1, 25), C (1, 25)	4 (4.7)
					PEN, NIT, RIF, ERY, GEN, TET, SXT, MUP (1, 25)		
					PEN (2, 50)		

ERY: erythromycin; PEN: penicillin; CIP: ciprofloxacin; TET, tetracycline; GEN: gentamicin; CLI: clindamycin; SXT: trimethoprim-sulfamethoxazole; MUP: mupirocin; RIF: rifampin; NIT: nitrofurantoin; SYN: quinupristin-dalfopristin; VAN: vancomycin.

## Data Availability

All data generated or analyzed during this study are included in this published article.
